# The effects of knee meniscectomy on the development of osteoarthritis in the patellofemoral joint 40 years following meniscectomy

**DOI:** 10.1007/s00590-019-02480-w

**Published:** 2019-07-10

**Authors:** Ioannis Pengas, William Nash, Angelos Assiotis, Kendrick To, Wasim Khan, Michael McNicholas

**Affiliations:** 1 Department of Trauma and Orthopaedics, Royal Cornwall Hospital, Truro, TR1 3LQ UK; 2 Department of Trauma and Orthopaedics, Guy’s Hospital, London, SE1 9RT UK; 3 Department of Trauma and Orthopaedics, St Mary’s Hospital, London, W2 1NY UK; 4 Division of Trauma and Orthopaedics, Department of Surgery, Addenbrooke’s Hospital, University of Cambridge, Cambridge, CB2 0QQ UK; 5 Department of Trauma and Orthopaedics, Aintree University Hospital, Liverpool, L9 7AL UK

**Keywords:** Knee, Meniscectomy, Patellofemoral joint, Tibiofemoral joint, Osteoarthritis, Long-term follow-up

## Abstract

Most knee osteoarthritis and meniscectomy studies focus on osteoarthritis in the tibiofemoral joint and ignore the patellofemoral joint. This study aims to assess the long-term effects of total meniscectomy on the patellofemoral joint. To our knowledge, this is the only study of osteoarthritis in the patellofemoral joint following meniscectomy that extends to a 40-year follow-up period. Twenty-two patients with osteoarthritis were evaluated at a mean of 40 years post-meniscectomy using standardised weight-bearing radiographs of the operated and non-operated knees. Patellofemoral joint osteoarthritis was diagnosed by the presence of osteophytes and joint space narrowing to less than 5 mm. Kellgren and Lawrence scores were calculated from the radiographs. Patellofemoral joint osteoarthritis and tibiofemoral joint osteoarthritis were correlated with International Knee Documentation Committee scores and range of movement measurements. A significant difference was observed between the operated and non-operated knees in terms of patellofemoral joint osteophyte formation. There was a significant difference in tibiofemoral joint Kellgren and Lawrence scores, International Knee Documentation Committee scores and range of movement measurements between knees with lateral facet patellofemoral joint space of < 5 mm and > 5 mm. This study shows an association between open total meniscectomy and patellofemoral joint osteoarthritis at 40 years following surgery. There was also an association between patellofemoral joint space narrowing in the lateral facet and tibiofemoral joint osteoarthritis. Possible causes include altered biomechanical loading patterns following meniscectomy as well as global processes within the knee.

## Introduction

The importance of patellofemoral joint (PFJ) osteoarthritis (OA) in relation to disability has been studied before [1]. It has been demonstrated that a standardised skyline view radiograph provides more information of the PFJ than a lateral view radiograph and has greater reproducibility [2–4]. Whilst a dedicated skyline view may be superior to a lateral view in depicting PFJ OA [5, 6], not all orthopaedic surgeons utilise this. Some studies suggest that up to 75% of clinicians opt not to use a skyline view in their assessment of knee pain [7].

**Fig. 1:**
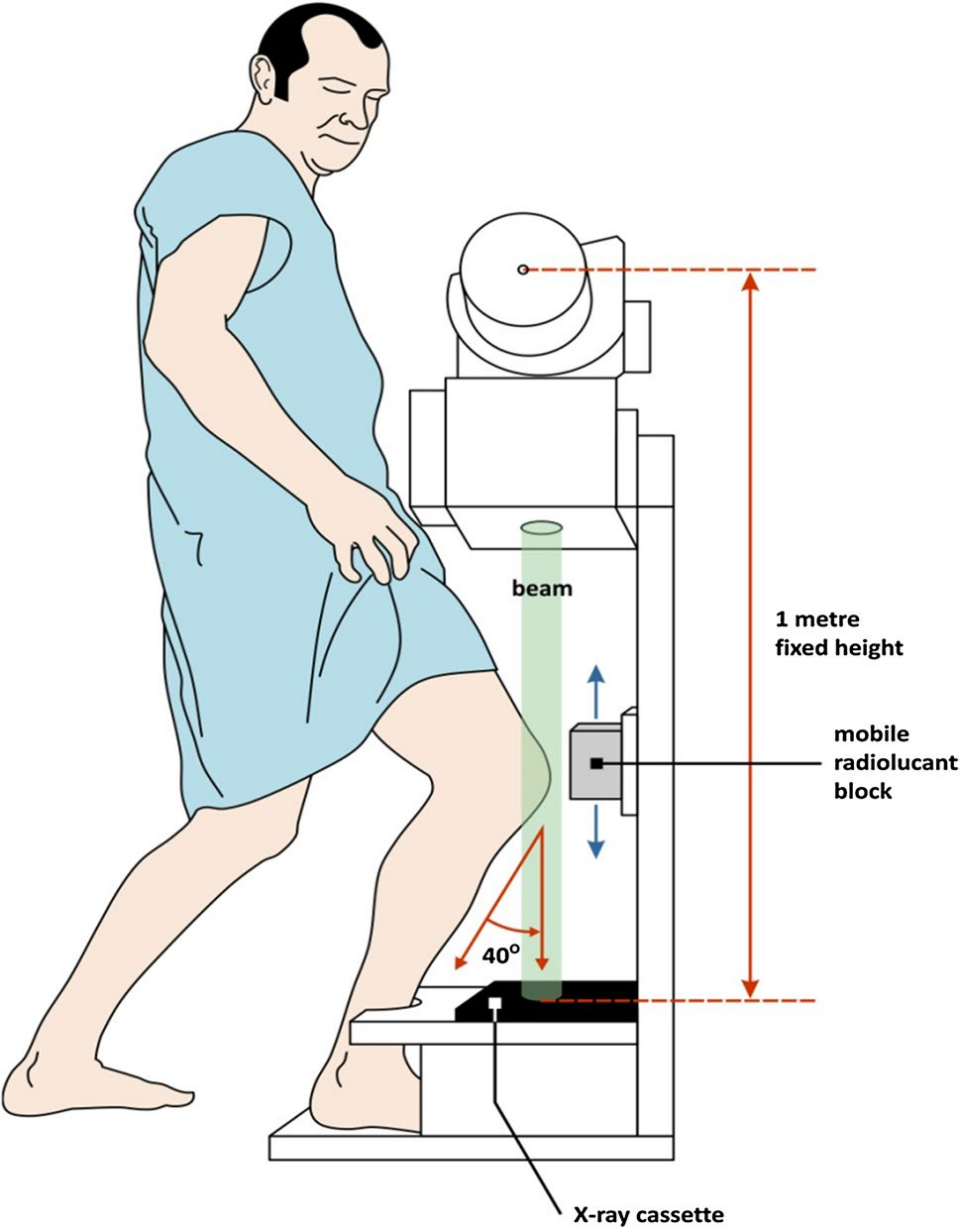
Skyline view of knee obtained in 40° of flexion

It has been well documented that meniscectomy is a significant risk factor for tibiofemoral joint (TFJ) OA [8–10], but there are fewer studies demonstrating its effect on the PFJ [11]. Some studies have shown that PFJ OA occurs most frequently in the lateral facet, with some reporting a frequency of 89% [12–14]. One particular study sought to further investigate the effects of joint space narrowing (JSN) to < 5 mm in the lateral facet [15]. In this study, we focus on the lateral facet. Our group previously published the effects of open meniscectomy on TFJ OA and patient-related outcome measures (PROMs) at a mean 40-year follow-up [16]. In this study, we are using the same cohort to assess the effects of meniscectomy on PFJ OA. A purpose-built device was validated [11] and used (Fig. 1) to assess this cohort. The same method of standardising a weight-bearing skyline view as per the aforementioned study was utilised.

## Methods

Under the auspices of the late Professor Iain Smillie, 313 adolescent patients underwent open total meniscectomy between 1960 and 1980. Of those patients, 100 who were confidently identified as having no other intra-articular knee pathology at the time of operation were reviewed at 17 years and 30 years post-operatively. Fifty-three patients were evaluated radiologically. This study represents the ongoing follow-up of the 53 patients at a mean of 40 years (33 to 50 years). Several patients were lost to the follow-up or excluded; five patients had died, seven had undergone a total knee replacement, six were lost to the follow-up, three declined to be assessed, and one was unable to attend due to multiple sclerosis. In addition, one patient who underwent lateral meniscectomy was unable to attend the clinic and completed the patient-related outcome measures (PROMs) questionnaires over the telephone; these data were excluded from subsequent statistical analysis. A total of 30 patients had objective and PROMs recorded at dedicated clinics. Eight patients had subsequently undergone interventions involving the contralateral knee after their 19th birthday; thus, the number of patients who had an intervention on one knee only at the time of review was 22.

All patients were reviewed by one assessor (IP), the range of movement (ROM) in both knees was recorded using a long-levered goniometer, and anterior tibial translation (sagittal laxity) was recorded with the rolimeter device (DJO, Vista, California) by averaging three consecutive readings of each parameter. Patient-reported outcomes were assessed using the International Knee Documentation Committee (IKDC) score. The PFJ was evaluated by standardised weight-bearing images at approximately 40° of flexion using a purpose-built device [11], as depicted in Fig. 1. The device projected a vertical beam of X-ray at a knee joint that was flexed to 40° with the subject in a standing position. The standardised weight-bearing skyline views of both the operated and non-operated knees were assessed in a darkened room and from the set distance of 60 cm without magnification equipment and scored in terms of the presence of osteophytes and the joint space (in each facet) measured by a ruler in increments of 1 mm. The KL score was utilised to quantify OA. Established OA was determined by the presence of osteophytes and its progression by JSN [17, 18].

### Statistical analysis

The data were analysed with SPSS 17.0 (SPSS Inc., Chicago) statistical package, where a *p* value of less than 0.05 was deemed to be statistically significant. A Shapiro–Wilk test was performed. Parametric data were analysed with a paired *t* test, whilst nonparametric data were subject to Wilcoxon signed-rank and the Mann–Whitney *U* tests. Kendall’s tau coefficient was then applied to correlate statistical dependence.

## Results

There was a significant difference between the operated and non-operated knees in the frequency of disease and the presence of osteophytes in the PFJ (*p* = 0.0015). The relative risk (RR) of developing radiologically diagnosed OA in the PFJ of operated versus non-operated knees was found to be 1.8 (95% CI 1.13–2.96).

A significant difference was seen between the operated knee and non-operated knee in terms of medial joint space; the space was measured to be 4.32 mm (± 2.88 mm) and 5.32 mm (± 2.06 mm), respectively (Table 1). In contrast, no significant difference was observed in the lateral joint space. There was a significant difference in the presence of osteophytes between operated and non-operated knees when the knees were globally assessed (1.41 ± 1.14 osteophytes and 0.45 ± (0.8) osteophytes, respectively), with less osteophytes seen in non-operated knees.

**Table 1:** PFJ JSN and the presence of osteophytes between operated and non-operated knees

	Operated knee—mean (± SD)	Non-operated knee—mean (± SD)	*p* value
Medial joint space (mm)	4.32 (2.88)	5.32 (2.06)	0.037
Lateral joint space (mm)	5.27 (2.75)	5.09 (2.41)	0.37
Osteophytes	1.41 (1.14)	0.45 (0.8)	0.0058

When the presence of osteophytes in the PFJ was grouped into the type of meniscectomy, no significant difference was identified between lateral and medial meniscectomy (Table 2). Out of 22 patients, seven underwent medial, nine underwent lateral and six underwent both medial and lateral meniscectomy. Osteophytes were observed in four out of seven, six out of nine and six out of six subjects, respectively.

**Table 2:** The presence of PFJ osteophytes and the type of meniscectomy performed

	Presence of osteophytes	*p* value
Medial meniscectomy	4/7, 57%	0.288
Lateral meniscectomy	6/9, 66%	
Medial and lateral meniscectomy	6/6, 100%	

The lateral facet of the PFJ in the operated knee was analysed, and mean differences in KL, IKDC and ROM results in the TFJ were recorded. A comparison was made between the PFJ with < 5 mm lateral facet joint space and PFJ with lateral joint space > 5 mm (Table 3).

**Table 3:** Lateral PFJ JSN and its association with TFJ parameters

	PFJ lateral facet joint space < 5 mm	PFJ lateral facet joint space > 5 mm	*p* value
TFJ KL score (mean ± 2xSD)	3.43 (2.36–4.50)	2.60 (0.003–5.20)	0.013
IKDC (mean ± 2xSD)	54.7 (41.4–68.0)	65.8 (43.1–88.5)	< 0.05
ROM (mean ± 2xSD)	118 (89–147)	129 (110–147)	< 0.00001

The results show an association between open total meniscectomy and PFJ OA at 40 years following surgery. There was also an association between patellofemoral joint space narrowing in the lateral facet and TFJ OA. Possible causes include altered biomechanical loading patterns following meniscectomy as well as global processes within the knee.

## Discussion

Evaluating patellofemoral joint PFJ OA can be challenging. To appropriately assess osteoarthritic change, we must bear basic principles in mind [17, 18]. For example, one scoring system, as proposed by Jones et al. [2], does not take joint space narrowing into consideration. Another study attempted to clarify the specificity of JSN as a measure of MRI-determined cartilaginous and osteoarthritic defects. A cut-off value of 5 mm, i.e. if joint space in either the lateral or medial facets of the PFJ was less than 5 mm, was found to have a high specificity for MR-detected cartilage defects. In addition, a separate study by the same group [4, 15] demonstrated that a PFJ with joint space of < 5 mm with the concomitant presence of osteophytes had sensitivity value and a positive predictive value of 90% and 95%, respectively, for MR-detected cartilage defects. It was shown that this sensitivity was reduced if the joint space was greater than 5 mm. Therefore, it is apparent that JSN to be less than 5 mm, with the presence of osteophytes, is diagnostic of OA in the PFJ.

In our study, a significant difference was observed between the operated and non-operated knees in terms of osteophyte presence and JSN. This indicates a correlation between meniscectomised knees and the development of osteoarthritis was illustrated by an observed relative risk of 1.8 (95% CI 1.13–2.96). In addition, there was no significant difference between the amount of joint space in the medial and lateral facets of the operated knees. However, the amount of lateral joint space demonstrated a significant effect on all measured patient outcomes. In addition, our analysis demonstrates a link between TFJ OA and PFJ OA following meniscectomy, as seen in the high KL score for the TFJ in operated knees with PFJ joint space of < 5 mm. There may be several explanations to this finding. Knee OA in general has been shown to be associated with hand OA suggesting that osteoarthritis could be a genetic, systemic as well as localised disease [19]. Activation of cytokine and protease cascades which act globally within the affected joint as well as systemically [20] could be one explanation, whilst altered biomechanical loading patterns post-meniscectomy could be another explanation [21]. Generalised OA in the post-meniscectomy knee could result in quadriceps weakness through disuse and may increase the risk of PFJ OA as well as TFJ OA.

## Conclusions

To our knowledge, this is the only study of osteoarthritis in the patellofemoral joint following meniscectomy that extends to a 40-year follow-up period. Our results suggest that meniscectomy increases the likelihood of subsequent patellofemoral joint osteoarthritis. This is associated with poorer functional outcomes. The underlying process appears to affect multiple parts of the knee joint. It is therefore important, when performing meniscectomies in young patients, to consider the implications of surgery on the long-term function of the knee joint. Consequently, there may be benefit in conducting long-term follow-up assessment in these patients.
